# Liquid embolic agents—a practical overview

**DOI:** 10.1186/s42155-026-00710-x

**Published:** 2026-05-29

**Authors:** Alessandro Cannavale, Lakshmi Ratnam, Andreas Mahnken, Alberto Alonso-Burgos, Enrique Esteban Hernandez, Carolina Lanza, Romaric Loffroy

**Affiliations:** 1 Vascular and Interventional Departmental Unit, University Hospital Policlinico Umberto I, Rome, Italy; 2 St George’s University Hospitals NHS Foundation Trust, London, UK; 3 City St. George’s University of London, School of Health & Medical Sciences, London, UK; 4 Diagnostic and Interventional Radiology and Nuclear Medicine, St. Josef-Hospital, University Hospital, Ruhr University Bochum, Bochum, Germany; 5 Radiology Department, Clinica Universidad de Navarra, Calle Santa Marta N. 1, Madrid, 28027 Spain; 6 Interventional Radiology, Radiology Department, Hospital Universitario de Sagunto, Valencia, Spain; 7 Department of Radiology, Fondazione Ca’ Granda Policlinico Di Milano, Milan, Italy; 8 Department of Vascular and Interventional Radiology, Université Bourgogne Europe, Image-Guided Therapy Center, ICMUB Laboratory, UMR CNRS 6302, François-Mitterrand Teaching Hospital, Dijon, 21000 France

**Keywords:** Embolisation technique, Sclerosants, Cyanoacrylates, Polymers

## Abstract

Liquid embolic agents (LEAs) have expanded the therapeutic options for complex vascular and non-vascular conditions. Tailored clinical assessment with dedicated procedure planning is critical in the selection of the most appropriate embolic to ensure procedural success and minimise complications. The LEAs, which range from sclerosant agents to glues and copolymers all have specific physical characteristics. Sclerosant agents are mainly selected for symptomatic vein diseases such as varicoceles and low-flow venous and lymphatic malformations (VM and LM), usually requiring multiple treatment sessions to achieve a satisfactory response. Glues and copolymers have completely different properties but may have overlapping indications ranging from haemorrhage control (gastrointestinal and solid-organ), treatment of visceral and renal aneurysms or pseudoaneurysms to type II endoleaks, arteriovenous malformations/fistulas, tumour devascularisation, and selective end-organ embolisation. The use of each agent varies according to the specific clinical scenario, and operator experience and preference. This review aims to provide a concise and practical overview of the use of LEAs.

## Background

Liquid embolic agents (LEAs) are new injectable materials used to permanently occlude blood vessels, especially in areas which cannot be easily reached by catheters or coils. They do not rely on the patient’s coagulation status and can be classified into different categories depending on their mechanism of action: sclerosant agents, in situ polymerisation agents (glues) and in situ precipitating agents (Ethylene–vinyl alcohol (EVOH)/Dimethylsulfoxide (DMSO) copolymers). This review aims to give an overview and practical tips on the most commonly used LEAs.

## Clinical assessment of patients

Patient evaluation is a longitudinal process beginning at the initial consultation and extending through treatment and follow-up. The interventional radiologist as a clinical specialist must review comorbidities, previous interventions, and current medications, ensuring that coagulation parameters, renal function, and systemic condition are optimised [1]. Communication with the referring team and patient is equally vital to balance therapeutic benefit against procedural risk [2].

Physical and clinical assessment provide insights that may influence treatment planning: ascites, jaundice, or vascular spiders may indicate portal hypertension and predict the presence of varices, whereas oedema or collateral circulation point toward altered venous return. Laboratory evaluation complements the clinical picture. As a general guide, haemoglobin levels lower than 8 g/dL, an INR above 1.5 or platelets below 50 000/µL may necessitate correction or selection of an agent less likely to induce distal migration or prolonged bleeding. Specific parameters will depend on local institutional and/or national guidelines, an example of which is the British Society of Interventional Radiology IR Procedure Bleeding Risk Guidance [3]. Pre-embolisation imaging defines the vascular architecture, identifies access routes, and allows an estimation of the expected embolic distribution.

The selection of the appropriate liquid embolic agent–such as cyanoacrylates, ethylene–vinyl alcohol copolymers, or sclerosants is a competitive decision driven by the underlying condition, the aim of the treatment, the individual patient´s condition and the mechanical or chemical features of the different liquids. In situations where any of several embolic agents would be appropriate, cost-effectiveness becomes a consideration. While specific costs vary in different countries and institutions, specific agents such as copolymers can significantly increase the procedural cost and may not be the best choice where a cheaper agent may provide the same result.

Factors to be considered in selection of agents include:

- Type of lesion: arteriovenous malformations (AVMs) with high-flow, multichannel architecture require fast-acting or deeply penetrating agents, while venous malformations (VMs) or slow-flow lesions allow more controlled deposition and prolonged injection times.
- Accessibility and reachability of the nidus determine whether a liquid agent can effectively penetrate the core of the lesion; limited access may necessitate adjunctive use of coils or plugs.
- Comorbidities such as allergies, arrhythmias, pulmonary hypertension, or lung fibrosis directly impact choice of embolic (e.g. no bleomycin in lung fibrosis), aesthetic management and the hemodynamic tolerance of embolisation.
- Coagulation state defines not only the procedural safety margin but also the agent’s polymerisation time and risk of distal migration; prolonged haemostasis times may contraindicate rapid-flow agents.

The clinical assessment serves as the decisive step that transforms technical feasibility into meaningful patient-centered therapy. It allows the interventional radiologist to move beyond a “one-size-fits-all” approach and to integrate anatomy, physiology, and patient-specific risks into the selection of the optimal embolic strategy. A thorough “look, touch, talk, think” approach ensures that each patient receives an individually tailored embolic therapy–optimising safety, efficacy, and long-term outcomes.

## Sclerosant agents

There are a variety of sclerotherapy agents available, with the most commonly used being sodium tetradecyl sulphate (STS), doxycycline, bleomycin and ethanol [4]. It is important to understand the physical properties of these agents, how they behave in different settings, and the various parameters which might alter their properties.

**Fig. 1:**
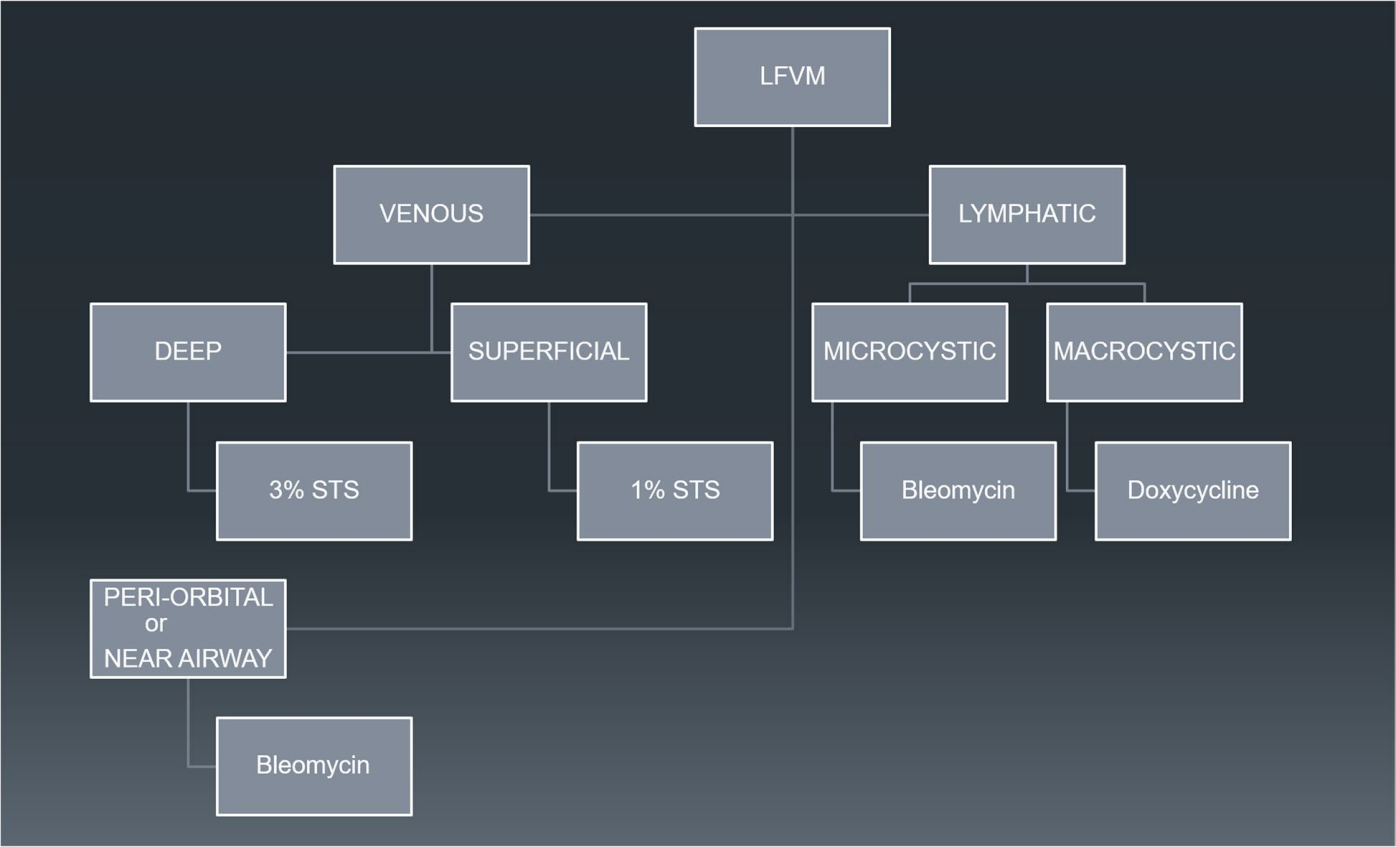
Algorithm for treatment of low flow vascular malformations (LFVM). STS: sodium tetradecyl sulphate

This section aims to give a general overview of common usage in sclerotherapy. STS can also be used in the treatment of varicose veins, varicoceles and in pelvic congestion syndrome. This has not been covered in this review. Complication rates can be reduced by having a good awareness of potential side effects, optimal preparation, volume titration and selecting the correct agent for each clinical scenario (Fig. 1).

### Sodium tetradecyl sulphate (STS)

#### Physical properties

STS is an anionic detergent which disrupts the normal architecture of the lipid bilayer of the cell membrane of the endothelial cells and denatures proteins such as clotting factors leading to fibrosis and vessel occlusion [5]. It is available in different concentrations and mixes homogenously with blood and results in uniform distribution after injection. To limit adverse sequelae, it can be used diluted (with contrast or saline), as a foam preparation, or using reduced concentrations for more superficial lesions to reduce skin ulceration and necrosis.

#### Technical tips

Foamed STS is prepared using the Tessari technique [6] with the constituents agitated using two 5 mL luer-lock syringes and a 3-way stopcock. The components used to create the foam are 1 mL Lipiodol: 2 mL 3% STS: 3 mL room air providing a total of 6 mL volume of foamed STS for injection. The foam is stabilised and rendered visible fluoroscopically by the Lipiodol.

### Doxycycline

#### Physical properties

Doxycycline is a type of tetracycline antibiotic which is relatively inexpensive and is readily available. It has a good side effect profile, and some studies have shown that there is a lower rate of progression to surgery for lymphatic malformations when treated with doxycycline compared to STS, and a quicker response. It works by inhibition of matrix metalloproteinases and cell proliferation, and suppression of vascular endothelial growth factor (VEGF) induced during angiogenesis and lymphangiogenesis. It also causes collagen and fibrin deposition, leading to formation of dense adhesions and fibrosis [7]. Macrocystic and mixed lymphatic malformations respond best to doxycycline sclerotherapy with poor response seen from microcystic lymphatic malformations. The most common complications are skin blistering or ulceration, and nerve injury [8].

#### Technical tips

In using doxycycline, cystic spaces are aspirated to dryness and doxycycline injected and left in situ. The volume injected should equate to half the aspirated volume. In order not to lose access to the drained space, where feasible, a small pigtail drain can be utilised. Recommended dose of doxycycline to be utilised is between 10 and 15 mg/kg in each session of treatment to minimise the risk of side effects as systemic absorption does take place even with intralesional treatment. The risks are dose related and are higher in infants and neonates [9].

### Use of doxycycline and STS together

Some authors have advocated the use of doxycycline and STS together. STS releases transmembrane lipoproteins from cell membranes, which increases cell membrane permeability and allows a secondary sclerosant (doxycycline) to better penetrate the cell and cause increased intracellular protein denaturation and cell death [10, 11].

The combined use has been described specifically in the treatment of neonatal lymphatic malformations. The method involves initial instillation of STS into the aspirated cavity. The STS is then removed and doxycycline injected and left in situ with the drain in place. The session is repeated on 3 successive days. Pain, swelling and skin necrosis are the most commonly reported side effects as with other sclerosant agents. A small number of cases of hypoglycaemia and, metabolic acidosis have been reported.

### Bleomycin

#### Physical properties

Bleomycin is an antitumoral agent that inhibits deoxyribonucleic acid (DNA), ribonucleic acid (RNA) and protein synthesis and has demonstrated local sclerosing effects on the endothelial cells of lymphatic walls and venous malformations [4, 12–16]. Bleomycin has been shown to produce less swelling than other sclerosants and is therefore preferred for treatment in areas where swelling is of particular concern, for example retro-orbital lesions and lesions near the airway [13]. Bleomycin has a specific side effect profile including risk of pulmonary toxicity (interstitial pneumonitis and eosinophilic hypersensitivity pneumonitis) and pulmonary fibrosis. Interstitial pneumonitis is dose dependent, whereas eosinophilic pneumonitis is not. Other potential risks are skin hyperpigmentation or discoloration and alopecia. It is important to have bleomycin protocols in place and that precautions are adhered to when using bleomycin (Table 1).

**Table 1:** Bleomycin precautions

Risk	Action	Precaution
Pulmonary fibrosis	1.Monitor bleomycin doses and adhere to maximum level per treatment 2.Avoid use of supplemental oxygen at time of bleomycin exposure. If needed use lowest oxygen concentration that is safe, use air if possible	**Pre-treatment** 1.Chest x-ray and lung function tests 2. Renal function tests
Skin marking	1. Avoid sticking anything adhesive to patient. If anything is stuck to the skin it should be very carefully removed using an adhesive solvent 2. Tie the endotracheal tube instead of taping 3. ECG dots should be placed in axillae or sole of feet so if they do mark, it would be inconspicuous 4. Intravenous cannulae should be secured with dressing not tape 5.Blood pressure cuff should be placed over cotton wool instead of directly on skin	**Post treatment** 1.Remind patients not to scratch skin for 48 h
Bleomycin disposal	1.Cytotoxic precautions for disposal of the drug should be carried out as per local pharmacy regulations	**Post treatment** 1.Document dose of Bleomycin and keep a record of cumulative dose over multiple treatments if applicable

#### Technical tips

Bleomycin is usually prepared in a cytotoxic laboratory within hospital pharmacies and is provided diluted in saline. It is important to be aware that there are different nomenclatures for dose in different parts of the world [16, 17]. In the UK, 1000 units is equivalent to 1 mg of bleomycin. For a session of sclerotherapy, no more than 15 000U is recommended in a single session with a lifetime dose limit of 80 000–100 000 U. The precise limits used vary from institution to institution. Cumulative dosage for individual patients must be documented to ensure this is not exceeded. For areas such as retro-orbital malformations, it is unusual to use more than 2 000 U in a single session, this would be used undiluted as 2 mL. However, for example for a session of bleomycin electrosclerotherapy (BEST), it is more common to use larger doses. Bleomycin can also be used as a foam preparation, especially for large lesions to obtain greater lesion coverage. The foam constituents comprise of 5 ml bleomycin (5000 U): 2 mL lidocaine 1%: 1 ml human albumin: 5 ml of room air which is foamed using the Tessari technique producing 13 mL of foamed bleomycin for injection.

### Ethanol

#### Physical properties

Ethanol functions as a sclerosant by denaturation of cellular proteins leading to damaged endothelium of the vascular wall and obliteration of the lumen. Almost all cells die within 5 s when exposed to 30% concentration of the sclerosant. Although it is very effective, the potential for very serious side effects is significant and it should be used with great care. Considering the good results from other sclerosants, the author does not recommend the use of alcohol for treatment of vascular malformations and would only advocate its use for sclerotherapy of cysts.

#### Technical tips

The use of ethanol is reported as being very painful, hence procedures are carried out under general anaesthesia. It is paramount to ensure that the ethanol remains within the injected cavity. This is ensured by using a pigtail drain where possible, and injecting contrast under fluoroscopy prior to injection of sclerosant. It is recommended that you do not exceed a dose of 1 ml/kg of alcohol or 50% of total volume aspirated from the cyst.

**Fig. 2:**
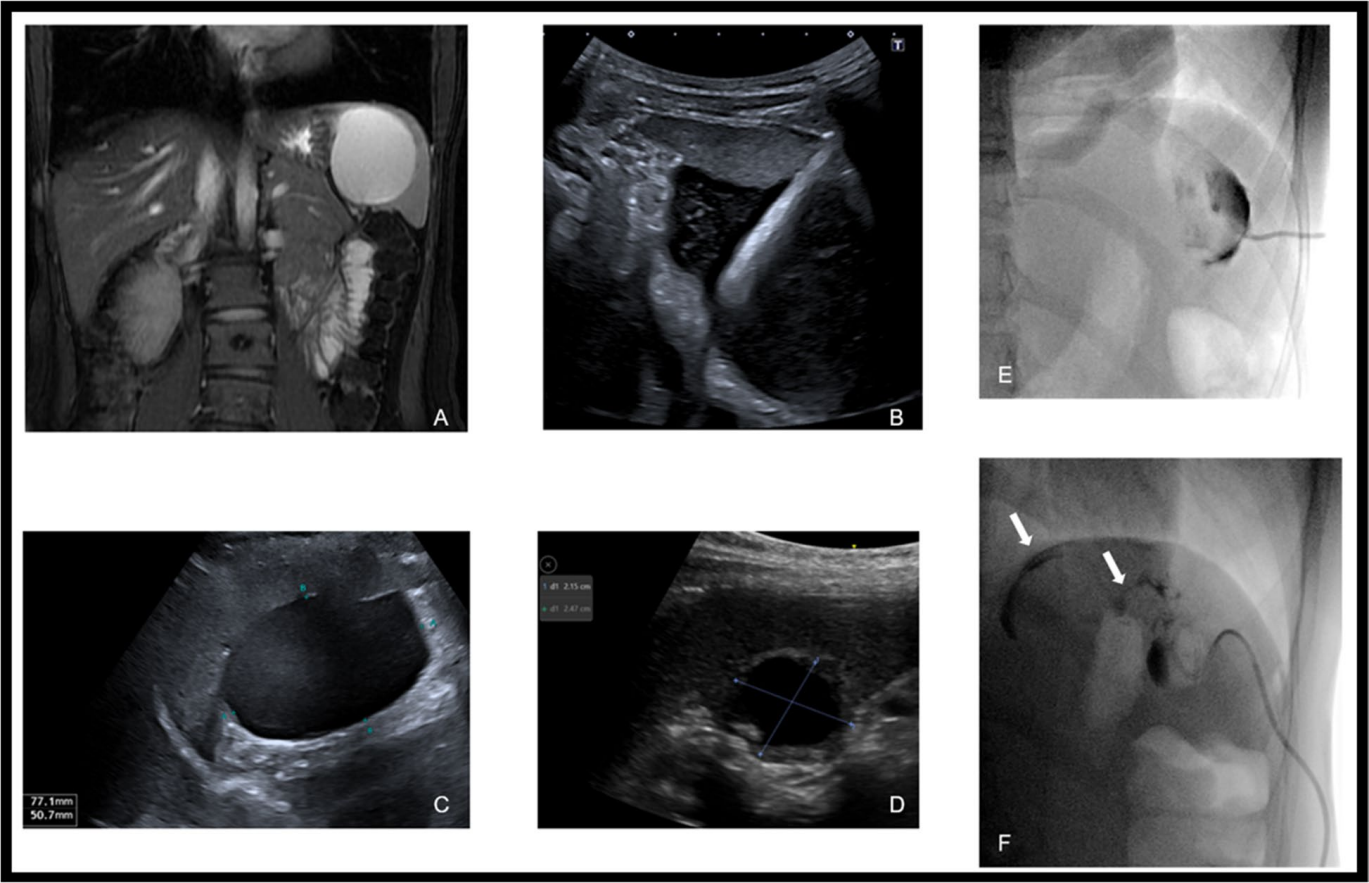
Different examples of sclerotherapy.** A** Large splenic cyst on coronal MRI. **B** Ultrasound guided insertion of Seldinger pigtail drain. **C** Cyst before treatment measuring 7.7 × 5.1 cm. **D** Cyst 4 months post one session of ethanol sclerotherapy measuring 2.2 × 2.5 cm. **E** Fluoroscopic contrast injection confirming drain within cyst before ethanol injection. **F** Different patient with splenic cyst – white arrows demonstrate contrast leaking out of cyst, thus unsafe for ethanol injection. Doxycycline was utilised instead in this patient

It is critical not to inject alcohol into retroperitoneum or intravascularly. Potential complications are alcohol intoxication, cardiovascular collapse and death [18]. Case examples are illustrated in Fig. 2.

## Glue/cyanoacrylate based agents

Following the empirical discovery of its haemostatic properties, cyanoacrylate was subsequently incorporated into the early experimental development of vascular embolisation techniques [19] and it is now one of the most commonly used liquid embolic agents in both peripheral and neuro-interventional practice [20].

### Physical properties

Cyanoacrylate is a monomer that remains relatively stable in its liquid form under dry conditions. Upon contact with water, hydroxyl groups, or weak bases–such as those present in the anionic environment of blood and the vascular endothelium–it undergoes rapid anionic polymerisation and instantaneous solidification. This achieves mechanical occlusion by occupying the vascular lumen and induces endothelial necrosis through an exothermic reaction accompanied by a pronounced inflammatory response, ultimately resulting in permanent vessel fibrosis within approximately one month after deployment [21]. Since the earliest formulations–Histoacryl® (N-butyl-2-cyanoacrylate; B. Braun, Melsungen, Germany) and subsequently TruFill® (N-butyl cyanoacrylate; Cordis, Miami Lakes, FL, USA)–the defining characteristic of cyanoacrylate-based embolic agents has been their intrinsic adhesiveness, which varies according to the specific chemical formulation [22, 23]. More recent agents (MagicGlue®, Balt, Montmorency, France; alpha-hexyl-cyanoacrylate) exhibit reduced adhesiveness and increased cohesiveness, thereby enhancing control and predictability [24]. In the following sections, cyanoacrylate-based liquid embolic agents will be described with reference to one of the most widely used preparations in Europe, N-butyl-cyanoacrylate with methacryloxy-sulpholane (NBCA; Glubran®2, GEM, Viareggio, Italy). This co-monomeric variant provides several advantages over earlier cyanoacrylate formulations: a slower polymerisation rate that facilitates handling and controlled delivery; a milder exothermic reaction (≈45 °C) and a more flexible final polymer that is less prone to fragmentation. NBCA undergoes immediate polymerisation upon contact with an anionic environment, typically within 1–2 s. Complete polymerisation, during which the adhesive attains its maximal mechanical strength, occurs within approximately 60–90 s [25, 26].

### Technical tips

The main limitation of cyanoacrylate-based embolic agents is the need for significant operator expertise, given the lack of universal guidelines or a standard formulation. Risks associated with the use of glue include catheter adherence to the occluded vessel and non-target embolisation by migration into distal vessels or reflux into proximal vessels [22]. The propagation of glue is influenced by the haemodynamic conditions at the catheter tip, modulation of NBCA polymerisation, and the injection technique. Table 2 outlines practical considerations for the safe and effective application of NBCA in embolisation procedures.

**Table 2:** Practical tips for use of glue

Before the glue injection	When aiming to achieve…	Under the condition of…	After the injection
Consider each clinical scenario independently: × Is the objective distal or proximal occlusion? × Is it a high-flow or low-flow situation? × Adjust the injection rate and polymerisation time of the NBCA:Lipiodol emulsion accordingly Use a compatible microcatheter × Internal PTFE lining × Polycarbonate-free hub Perform injection test with contrast medium Prepare the NBCA: Lipiodol emulsion in advance 3 to 5 mL syringes for injection	**PROXIMAL OCCLUSION** × Ratio glue:Lipiodol 1:1/1:2 × D5 flush only the catheter dead space	**HIGH FLOW** × Coil-assisted “scaffold technique” × Faster injection, shorter polymerisation × High NBCA:Lipiodol ratio	× No urgency in catheter withdrawal × Quick and dry “snap” pull for removal
	**DISTAL OCCLUSION** × Ratio > 1:3 × D5 flush catheter and vascular bed thoroughly	**LOW FLOW** × Slower injection, prolonged polymerisation × Low NBCA:Lipiodol ratio	

Key technical steps include:

- **Catheter preparation:** flush dead space with 5% dextrose to prevent intraluminal polymerisation.
- **Catheter choice:** use fluoropolymer-lined (polytetrafluoroethylene, PTFE) catheters to prevent obstruction or even catheter fracture during withdrawal.
- **Mixture preparation:** cyanoacrylate is radiolucent. It must therefore be emulsified with ethiodised oil (Lipiodol® Ultra Fluid; Guerbet, Aulnay-sous-Bois, France) to render it radioopaque [11]. The NBCA:Lipiodol® emulsion is prepared simply. Lipiodol® is drawn into one syringe and cyanoacrylate into another. The syringes are then connected via a three-way stopcock. Approximately twenty rapid back-and-forth passes are performed to achieve adequate mixing. This procedure must be executed swiftly, as the mixture can compromise the integrity of the stopcock, typically composed of polycarbonate [13].
- **From the injection standpoint:** the primary technical consideration is the mechanical interface between the catheter tip and the embolic agent. Curved tips may transmit lateral forces to the vessel wall which will increase the likelihood of retrograde reflux and catheter entrapment upon retrieval due to adhesion of polymerised glue to the catheter. Straight-tipped microcatheters are therefore preferred, allowing greater control over injection dynamics and reducing the risk of complications. For injection, the use of 3- or 5-mL syringes is recommended to ensure greater control during administration.

**Fig. 3:**
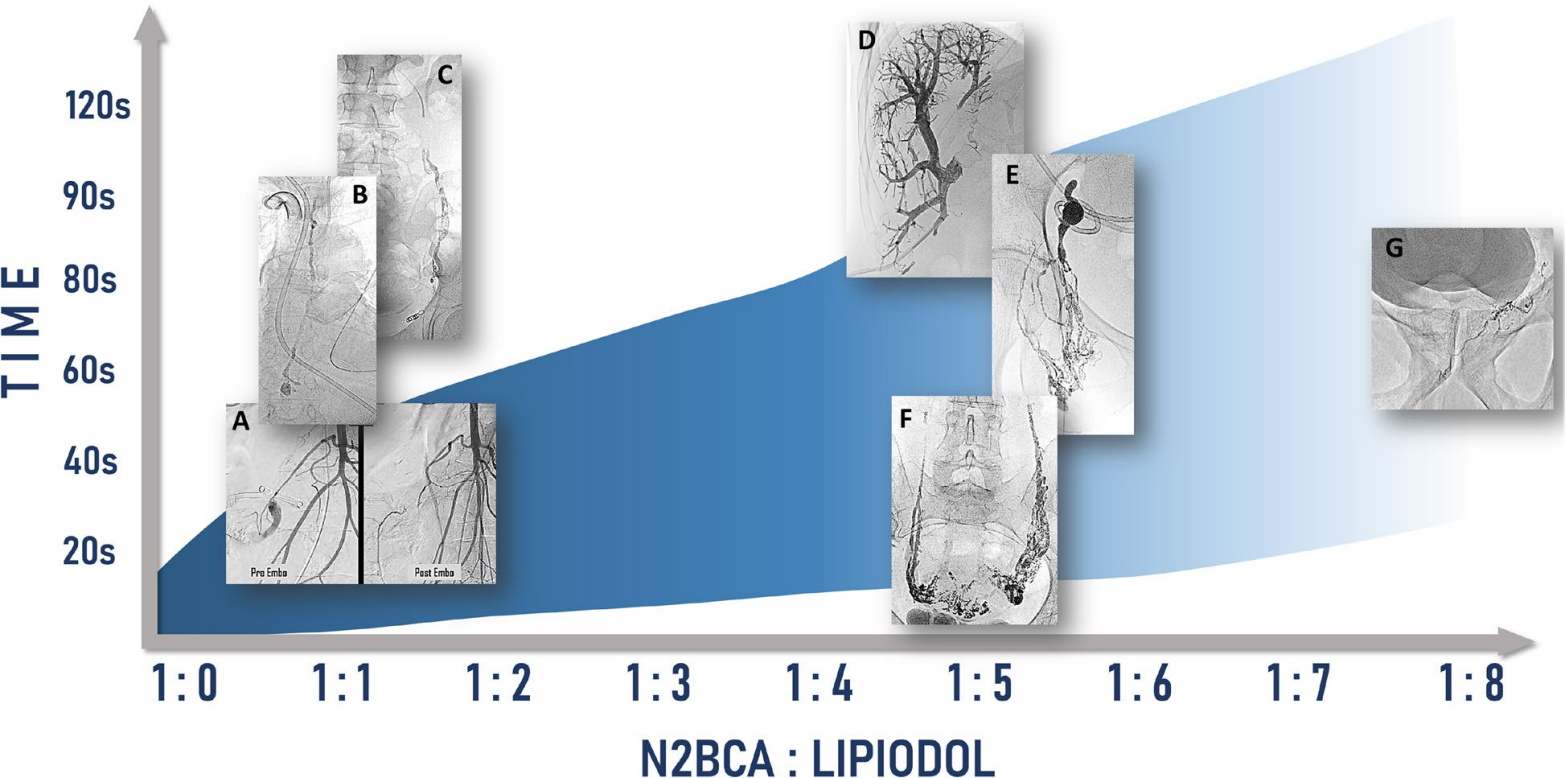
Schematic representation of the modification in NBCApolymerisation kinetics (onset and duration) according to the NBCA:Lipiodol emulsion ratio, with illustrative examples of different clinical scenarios for each ratio.** A** Traumatic bleeding. **B** Thoracic duct embolisation. **C** Varicocele. **D** Portal embolisation. **E** Lymphatic leak transnodal embolisation. **F** Pelvic congestion syndrome. **G** Prostatic artery embolisation

The polymerisation kinetics can be precisely modulated by:

- **Dextrose flushing:** a higher dextrose volume creates a non-anionic environment at the catheter tip, thereby preventing premature polymerisation [27].
- **Adjusting NBCA:Lipiodol® ratio:** increasing the Lipiodol® content delays the onset of polymerisation and prolongs the overall setting time [25, 28]. Figure 3 shows how the kinetics of the NBCA:Lipiodol® emulsion vary. It highlights differences in the onset and duration of polymerisation. It also outlines its use across a range of clinical applications [25].

Cyanoacrylate migration can also be controlled through the simultaneous use of mechanical embolic agents, such as coils or plugs (Table 2) [26, 29].

### Clinical outcomes

NBCA is used across a wide range of clinical settings. These include arterial embolisation, such as treatment of peripheral arteriovenous malformations, preoperative tumour embolisation, and haemorrhage control. Venous applications include embolisation of the portal vein, gonadal veins, and pelvic varices. It is also used in percutaneous procedures, including the treatment of type II endoleaks and pseudoaneurysms. Each indication should be assessed individually. Technical considerations must be tailored to the specific objectives of each case [21, 22, 27].

A “1 to 1” NBCA:Lipiodol® ratio– or higher–yields a consistency similar to a “liquid coil,” ideal for embolisations where distal migration must be minimised and immediate polymerisation is required. This mixture can be prepared rapidly and functions independently of the coagulation status. It is therefore particularly suited to the management of life-threatening haemorrhage, including trauma, pelvic fractures, postpartum haemorrhage, and gastrointestinal bleeding. It is also especially appropriate for use in anticoagulated patients [21, 27].

The same ratio is particularly effective for thoracic duct embolisation [30], leveraging both the adhesive and sclerosing properties of the cyanoacrylate mixture. It can also be applied in the venous system for the treatment of varicoceles, with excellent outcomes. Beyond the adhesive’s capacity to penetrate collateral territories, its local inflammatory and sclerosing effects promote thrombosis in potential collateral vessels [26, 31].

From the perspective that lower NBCA:Lipiodol® ratios (1 to 5 or 1 to 6) delay polymerisation, this approach is particularly advantageous for portal vein embolisation–allowing distal access from proximal sites, and for pelvic congestion syndrome, which requires extensive venous embolisation due to high collateralisation; and in transnodal lymphatic embolisation [30, 32–34].

More dilute preparations, such as a “1 to 8” ratio, can also be employed for embolisation of the prostatic artery. In this setting, cyanoacrylate has demonstrated efficacy comparable to that of embolic particles, while offering procedural advantages in terms of reduced procedure time. It is also useful in situations where particle embolisation may be contraindicated [35, 36].

The use of liquid embolic agents in the clinical management of haemorrhoidal disease remains a matter of ongoing debate [37, 38]. NBCA has been employed with favourable clinical outcomes and an acceptable safety profile in the embolisation of gastrointestinal haemorrhage. However, within the specific clinical context of haemorrhoidal disease, to the best of our knowledge, no studies have yet been published on this subject. Nevertheless, isolated reports suggest the potential use of this agent as an embolic material [39].

Two key considerations must, however, be addressed at the outset. First, effective treatment requires distal embolisation of the various arterial inflows arising from the superior rectal artery. Second, the intrinsic inflammatory response associated with NBCA as an embolic agent must be taken into account. Accordingly, careful determination of the NBCA:Lipiodol® ratio is essential, not only to ensure adequate distribution to the distal vascular bed but also to modulate the degree of procedure-related inflammation.

This consideration implies a clinical scenario in which early outcomes may be characterised by symptoms of proctitis secondary to the inflammatory response. Finally, there is a clear need for medium- and long-term studies using an appropriate animal model, likely not porcine, given the relatively limited collateralisation of rectal vascularisation compared with humans, to better define the true risk of ischaemia/necrosis and late stenotic complications [40].

## Copolymers

### Physical properties

Non-adhesive liquid embolic agents (NALEAs) used outside the neurovasculature are dominated by two copolymer families: ethylene–vinyl alcohol (EVOH) systems (e.g., Onyx™ and Squid™) and iodine-bearing copolymers (e.g., PHIL™). Both are dissolved in dimethyl sulfoxide (DMSO) and injected through DMSO-compatible microcatheters. On contact with blood, DMSO diffuses away and the polymer precipitates into a cohesive, spongy cast that occludes the target vessel. [41, 42].

#### Radiopacity and artifacts differ

EVOH products suspend micronised tantalum for X-ray visibility. As tantalum is a particulate material, vials must be vigorously mixed (≈20 min on a dedicated mixer) and visibility can decline during long injections as the tantalum sediments in the syringe or microcatheter. This also contributes to microcatheter blockage and more pronounced CT beam-hardening artefacts post-procedure [43]. Iodine-based systems such as PHIL covalently bind iodine within the polymer, eliminating the need for shaking and providing more homogeneous, time-stable radiopacity. The covalent iodine reduces CT and cone-beam CT artefacts compared with tantalum-based agents which is advantageous for example when evaluating post-endovascular repair sacs or tumor beds [44].

#### Rheology and compositions

EVOH formulations are available in different viscosities (e.g., Onyx 18 vs 34 and Squid 12 vs 18 vs 34) to balance penetration versus reflux resistance [45–47]. Iodine-based agents similarly come in graded concentrations (e.g., PHIL 25/30/35) with higher-concentration/viscosity mixtures preferred for high-flow lesions or when building a rapid plug [48]. Across both families, the non-adhesive nature allows prolonged, stop-start injections and “plug-and-push” techniques without gluing the catheter in place– an important practical distinction from cyanoacrylate glues [47].

### Technical tips

#### Where they are used

In the body, EVOH and iodine-based copolymers are used for acute arterial haemorrhage (gastrointestinal and solid-organ), visceral and renal aneurysms or pseudoaneurysms, type II endoleaks, AVMs/AVFs, tumour devascularisation, and selective end-organ embolisation (e.g., symptomatic renal angiomyolipoma). Their cohesion and deep-penetration help treat lesions unreachable by coils/particles. They also remain effective in coagulopathic patients as vessel occlusion relies on the formation of a polymer cast rather than clot formation [49–53].

#### Microcatheter and solvent essentials

DMSO-compatible microcatheters and syringes should always be used as incompatibility risks catheter damage and/or obstruction. Before connecting the embolic syringe, pre-fill (wet) the microcatheter dead space with DMSO to avoid premature precipitation and air contamination (“wet-to-wet” connection). Inject DMSO slowly to minimise vasospasm and patient discomfort [43, 46].

#### Mixing and preparation

Onyx and Squid require continuous mixing for ≥ 20 min to obtain uniform tantalum suspension; for prolonged cases with long injection times, periodic syringe exchange or remixing may be necessary to maintain visibility. PHIL arrives in pre-filled syringes and does not require shaking, which can shorten preparation time in emergencies [44, 45].

#### Plug-and-push with reflux control

**Fig. 4:**
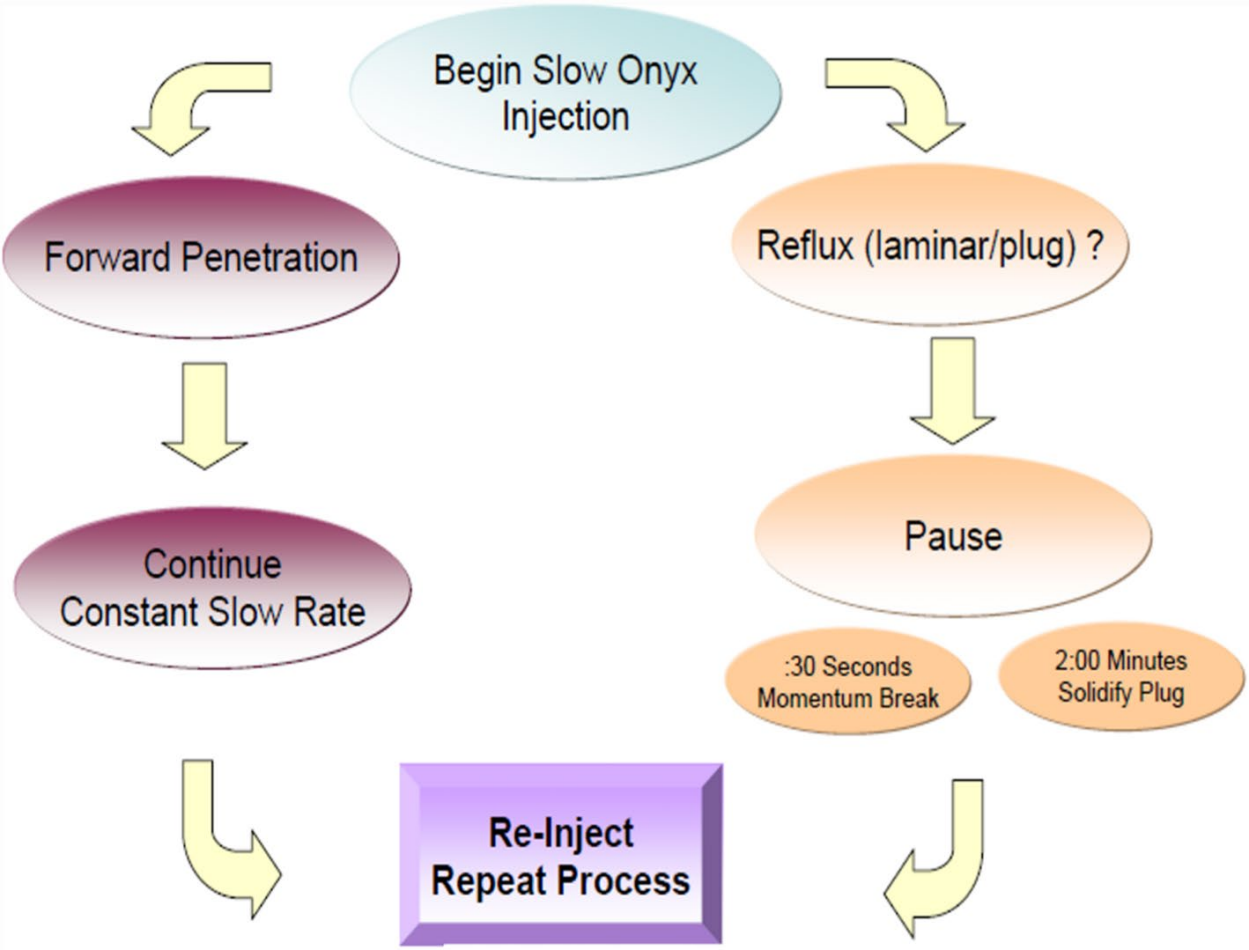
Plug-and-push technique for injection of EVOH-based embolic agents

For both EVOH and PHIL, the technique frequently employed is to create a short reflux “plug” at the catheter tip, then gently advance the cast (“lava-like” flow) through the nidus or into the aneurysm sac. Intermittently pausing injection allows further precipitation and cast formation (Fig. 4). Reflux is controlled by choosing higher-viscosity formulations, using dual-lumen balloon microcatheters (for temporary flow arrest), or briefly wedging the catheter tip. Blank roadmap images are used to monitor progression of the cast; avoiding long uninterrupted injections that might outpace DMSO diffusion or worsen reflux [42, 45].

#### Adjuncts and sequencing

EVOH/PHIL can be combined with coils or plugs (“plug-and-paint”: line the sac/neck with coils, then seal interstices with polymer) to reduce reflux and polymer volume, and secure wide-neck aneurysms or endoleak sources. Embolisation with particles proximally can be considered for flow reduction before deep polymer penetration in diffuse haemorrhage [52]. When using EVOH with coils, it is important to be aware of possible DMSO–polymer interactions with the coatings of some coils (as outlined in individual device IFU).

#### Imaging considerations

Greater streak artefact can be expected with tantalum-based casts on CT scan; when post-EVAR sac surveillance or parenchymal evaluation is critical, iodine-based agents may simplify interpretation. Conversely, if you anticipate prolonged injections in small tortuous feeders, ensure adequate fluoroscopic parameters and consider the radiopacity profile of your agent and formulation [54–56].

Tips and pitfalls are summarised respectively in Tables 3 and 4.

**Table 3:** Essential technical tips for copolymer embolisation

Category	Key tip/Best practice
Preparation	Always use DMSO-compatible microcatheters and syringes; prime with DMSO (“wet-to-wet”) to prevent premature polymerization
Mixing & handling	Shake EVOH (Onyx™) for ≥ 20 min to maintain uniform tantalum suspension. Iodine-based copolymers (PHIL™) are ready-to-use – no shaking required
Injection technique	Build a short 3–5 mm reflux plug, then inject slowly and intermittently (“plug-and-push”) under roadmap guidance
Flow management	For high-flow or large feeders, use balloon-assisted or wedged-catheter delivery and select higher-viscosity polymer formulations
Pain control & safety	Inject DMSO slowly (≤ 0.1 mL/s) to minimize vasospasm; premedicate with analgesics or intra-arterial lidocaine as needed. Wait 1–2 min before catheter withdrawal

*DMSO *Dimethyl sulfoxide, *EVOH *Ethylene vinyl alcohol

**Table 4:** Common pitfalls in copolymer embolisation and how to avoid them

Pitfall	Consequence	Prevention strategy
Using non-DMSO-compatible devices	Catheter rupture or polymer precipitation	Verify device compatibility; follow IFU strictly
Skipping DMSO priming	Air or clot formation within catheter	Always perform wet-to-wet DMSO flush before injection
Inadequate mixing of EVOH	Loss of radiopacity, catheter clogging	Shake continuously for ≥ 20 min and remix if idle
Over-injection without pauses	Reflux, non-target embolisation, catheter entrapment	Slow intermittent injection; limit reflux to ≤ 1 cm
Ignoring flow dynamics	Polymer migration or incomplete occlusion	Match viscosity to flow; use balloon occlusion if needed
Rapid DMSO injection	Severe pain, vasospasm, vessel injury	Keep injection ≤ 0.1 mL/s; use local analgesia
Premature catheter withdrawal	Entrapment or vessel injury	Wait 1–2 min post-injection; rotate gently while withdrawing

*DMSO *Dimethyl sulfoxide, *IFU *Instructions for use

### Clinical outcomes

#### Acute haemorrhage and peripheral haemostasis

Multiple series document high technical and clinical success for EVOH in non-neuro bleeding [45, 54, 56]. In one peripheral/visceral cohort of 50 patients undergoing Onyx embolisation for haemostatic or non-haemostatic indications, technical success was 100% and primary clinical success 100%, with minimal complication rates [45]. Another review demonstrated clinical effectiveness of EVOH in peripheral haemorrhage broadly [42]. Iodine-based copolymers show comparable outcomes. In some non-neuro bleeding cohorts treated with PHIL, technical success approaches 97% with low non-target embolisation and acceptable DMSO-related pain [48]. Although head-to-head comparative data are limited, these agents are increasingly considered safe and effective in the extra-cranial space.

#### Endoleaks (type II)

**Fig. 5:**
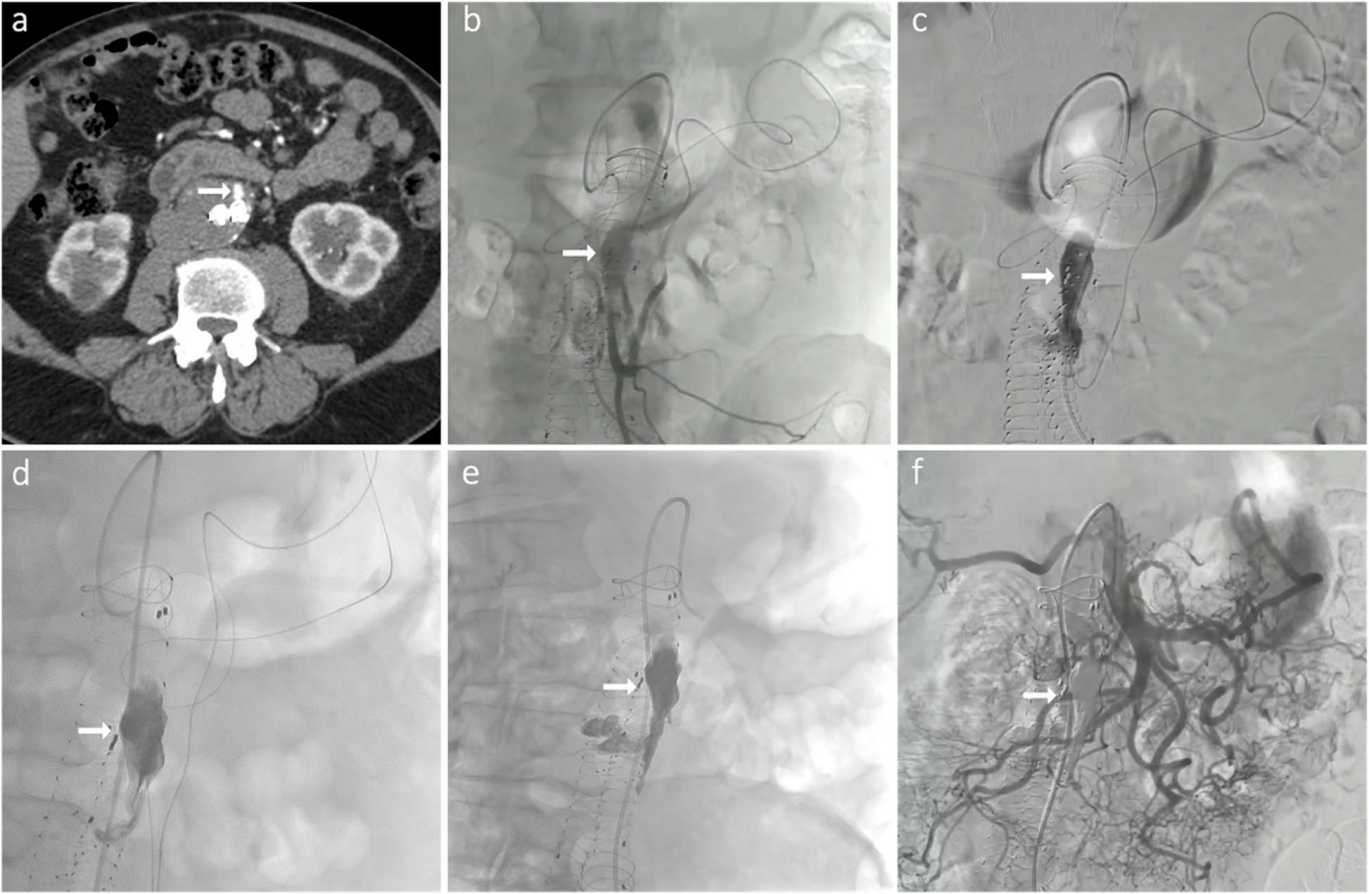
Type IIa endoleak with growing aneurysmal sac in a 75-year-old patient with previous history of EVAR.** A** Abdominal CT scan showing type IIa endoleak (nidus) by retrograde opacification of the sac via the inferior mesenteric artery (IMA). **B** Selective angiogram of the IMA through a 2.0-Fr microcatheter via the Riolan arcade showing type IIa endoleak. **C** Placement of the microcatheter into the aneurysmal sac. **D** Embolization with Onyx 18 into the sac. **E** Complete filling of the nidus with the copolymer. **F** Final angiogram showing complete exclusion of the endoleak with preservation of the patency of the main IMA

EVOH-based NALEAs are commonly used for type II endoleak embolisation due to their ability to permeate the nidus of retrograde feeders (Fig. 5). Clinical series report high success rates [47]. For example, in a comparison of Onyx vs coils for type II endoleaks, patients treated with Onyx had lower rates of reintervention (19% vs 55%) over mean follow-up of ~ 57 months [51].

#### Renal angiomyolipoma (rAML) and other organ-specific (liver, prostate)

Selective embolisation is effective for haemorrhage control and prevention in rAML. While many series use diverse agents (coils, glue, particles), EVOH is frequently included as a stand-alone or adjunct, leveraging its deep penetration and durable occlusion; contemporary cohorts show durable mid-term outcomes after trans-arterial embolisation [55–57]. Iodine-based copolymers are increasingly reported in similar roles, but high-quality comparative data remain limited [50]. Few data exist on the promising role of copolymers for portal vein embolisation or treatment of benign prostatic hyperplasia [53, 58].

#### Safety profile and complications

Across both families, non-target embolisation rates are low when super-selective techniques and reflux control strategies are used. DMSO can cause transient vasospasm, flushing or pain which is mitigated by slow injection and appropriate analgesia. Catheter entrapment is uncommon with non-adhesive agents but can occur if the cast envelops a deeply wedged tip, hence the importance of short pauses, controlled reflux, and awareness of dead space. Artefact considerations (tantalum vs iodine) are chiefly an imaging-follow-up issue rather than a safety concern [41, 42].

## Discussion

Liquid embolic agents represent an essential tool in the armamentarium of interventional radiology. In comparison to traditional embolic agents, such as coils and plugs, a significant advantage of LEAs is their ability to penetrate the distal peripheral vasculature, to regulate the penetration depth and provide effective vessel occlusion, regardless of the patient’s coagulation status [59]. The drawback of these agents on the other hand, is the long learning curve required to avoid technical complications [60].

The sclerosant agents discussed are sodium tetradecyl sulphate, doxycycline, bleomycin and ethanol. These are indicated for the treatment of symptomatic low-flow venous and lymphatic malformations (VMs and LMs). A systematic review found limited evidence supporting superiority of any agent, with efficacy across the different agents ranging from 70 to 100% [4]. Reported complication rates are mostly self-limiting with ethanol sclerotherapy being associated with most high-grade complications such as skin necrosis, and therefore not recommended for routine sclerotherapy. Sclerosants can also be utilised for the treatment of recurrent cysts and seromas.

Cyanoacrylate glues are indicated in the management of bleeding, with a unique feature of being effective in coagulopathic patients [61–63]. Although widely used for embolisation in emergency settings, their effective and safe use has also been documented in elective procedures, including prostatic artery embolisation, portal vein and bronchial artery embolisation [64–66]. NBCA as an agent is rapid and effective in its occlusive power, but a pitfall is the experience needed to avoid technical complications including microcatheter entrapment and non-target embolisation [67]. New developments such as detachable-tip microcatheters, and modification of NBCA–Lipiodol ratios enable the controlled delivery of the embolic agent and reduce catheter entrapment rates.

Non-adhesive polymers, particularly EVOH copolymers (Onyx, Squid), have significantly expanded the therapeutic possibilities in both cerebral and extracranial procedures. Their slow precipitation mechanism allows controlled injection, known as “magma-like” flow, making them especially suitable for AVMs, hypervascular tumors, and endoleaks. Multiple publications have described their high rates of nidus penetration and durable occlusion [68–70]. The range of different formulations available with varied viscosities (e.g., Squid 12) has been shown to improve distal arterial penetration, improving results in micro-nidus embolisation [70].

In conclusion, LEAs selected based on lesion characteristics, agent properties, patient status and operator experience/preference are a valuable tool with good evidence across a multitude of indications. This review provides a framework outlining properties, indications, advantages and disadvantages of the various LEAs and a summary of potential complications and clinical outcomes coupled with expert tips for use.

In the future, larger comparative trials can refine existing treatment algorithms as well as evaluate emerging formulations and new embolic agents, to support more reproducible outcomes.

## Data Availability

All data generated or analysed during this study are included in this published article.

## Abbreviations

AVF: Arteriovenous fistula
AVM: Arteriovenous malformation
BEST: Bleomycin electrosclerotherapy
DMSO: Dimethyl sulfoxide
DNA: Deoxyribonucleic acid
D5: Dextrose 5%
EVAR: Endovascular aneurysm repair
EVOH: Ethylene–vinyl alcohol
IFU: Instructions for use
LEA: Liquid embolic agent
LM: Lymphatic malformation
NALEA: Non-adhesive liquid embolic agent
NBCA: N-butyl-cyanoacrylate
PTFE: Polytetrafluoroethylene
rAML: Renal angiomyolipoma
RNA: Ribonucleic acid
STS: Sodium tetradecyl sulphate
VEGF: Vascular endothelial growth factor
VM: Venous malformation
